# Molecular Insight into the Possible Mechanism of Drag Reduction of Surfactant Aqueous Solution in Pipe Flow

**DOI:** 10.3390/ijms22147573

**Published:** 2021-07-15

**Authors:** Yusei Kobayashi, Hirotaka Gomyo, Noriyoshi Arai

**Affiliations:** Department of Mechanical Engineering, Keio University, 3-14-1 Hiyoshi, Kohoku-ku, Yokohama 223-8522, Japan; h.gomyo8@gmail.com (H.G.); arai@mech.keio.ac.jp (N.A.)

**Keywords:** drag reduction, surfactant molecules, self-assembly, coarse-grained molecular simulation

## Abstract

The phenomenon of drag reduction (known as the “Toms effect”) has many industrial and engineering applications, but a definitive molecular-level theory has not yet been constructed. This is due both to the multiscale nature of complex fluids and to the difficulty of directly observing self-assembled structures in nonequilibrium states. On the basis of a large-scale coarse-grained molecular simulation that we conducted, we propose a possible mechanism of turbulence suppression in surfactant aqueous solution. We demonstrate that maintaining sufficiently large micellar structures and a homogeneous radial distribution of surfactant molecules is necessary to obtain the drag-reduction effect. This is the first molecular-simulation evidence that a micellar structure is responsible for drag reduction in pipe flow, and should help in understanding the mechanisms underlying drag reduction by surfactant molecules under nonequilibrium conditions.

## 1. Introduction

In the 21st century, soft-matter rheology is recognized as a vitally important field with applications to engineering (e.g., food [1,2], cosmetics [3], medical materials [4]), biology (e.g., strain hardening of fibrin [5] and the motion of motor proteins [6,7]), and the global environment (e.g., mantle flow [8,9] and the origin of life [10,11]). However, the behavior of soft matter is difficult to understand because it encompasses phenomena on multiple spatiotemporal scales, and rheology involves the study of inherently nonequilibrium phenomena. Thus, there are major barriers to understanding either separately, much less in combination; soft-matter rheology remains a challenging subject. The pioneering work of De Gennes [12], and Doi and Edwards [13,14,15] in the late 1970s sparked interest in explaining the rheological properties of entangled polymer melts by advanced physical modeling. Their “tube model’’ was able to explain, to a certain extent, the relaxation dynamics of entangled polymers. However, quantitative tube-model predictions for complex polymers, including branched and di-block copolymers and blends, are still not possible because they involve molecular details below tube length. In order to predict and understand the rheological properties of actual soft matter, it is essential to incorporate the properties of molecules.

In recent years, computer simulations have been successfully used to reproduce the behavior of molecules inside complex soft matter and to clarify the source of their rheology [16,17]. For example, theoretical expressions describing the plateau moduli of slip-link and slip-spring models were proposed by Uneyama and Masubuchi [18]; reasonable agreement between their theory and simulations has been confirmed. Numerical simulation [19] showed that shear can promote the crystallization of colloidal star polymers in the vicinity of their glass transition, and that a transition from a bcc to an fcc structure can occur.

One of the major unsolved problems in soft-matter rheology is the origin of drag reduction caused by polymers or surfactants, the so-called Toms effect [20], for which a definitive theory has not yet been constructed because molecular-scale details remain unknown. Nevertheless, the Toms effect has many industrial and engineering applications, including district cooling systems, firefighting, and the pipeline transportation of natural gas, water, and crude oil. Since Toms first discovered the effect using polymer solutions [20], extensive and continuing research on drag reduction by additives has been conducted via numerical simulations [21,22,23,24] and experiments [25,26,27,28]. Among various drag-reducing agents, surfactants have an advantage over polymers from a practical standpoint because surfactant molecules are able to reform micelle structures even after mechanical degradation (except under extreme shear conditions) [29]. The relation between the viscosity behaviors of surfactant aqueous solutions and the formation of micelles was investigated through coarse-grained molecular-dynamics simulations [30,31,32,33,34], but these studies did not provide evidence regarding the frictional coefficient of pipes.

Several possible mechanisms of turbulent drag reduction have been proposed and are summarized in recent reviews [35,36,37]. In particular, many previous works suggested that a close relationship exists between the viscoelastic behavior of micellar structures and the Toms effect. Nevertheless, despite extensive research conducted on the topic, no universally acceptable mechanism has yet been identified. This is partly because of the usual multiscale problem in soft-matter systems, but also because the direct experimental observation of self-assembled structures of surfactants under nonequilibrium (e.g., turbulent-flow) conditions is an extremely challenging task. In addition, turbulent flow is intrinsically difficult to understand because of the large number of parameters it involves. Hence, most studies provide only phenomenological explanations under certain conditions; a fundamental understanding of the relation between self-assembled structures and the associated drag reduction is still lacking.

In this study, using large-scale dissipative particle dynamics simulation, we study the relationship between the self-assembly of surfactant molecules and their flow properties under pipe flow. Our goal is to understand the mechanism of turbulence suppression in a surfactant aqueous solution from a molecular viewpoint. The structures and distributions of micelles under turbulent flow are investigated, and the necessary conditions to obtain the drag-reduction effect are determined.

## 2. Model and Methods

### 2.1. Dissipative Particle Dynamics (DPD) Method

We employed the dissipative particle dynamics (DPD) [38,39,40] method to study the turbulent drag-reduction effects of a short-chain surfactant aqueous solution in pipe flow using inhouse code. The DPD method can simulate millisecond time scales and micrometer length scales because only the motion of coarse-grained particles (i.e., groups of atoms or molecules) is simulated. To date, many previous studies [41,42,43,44] using the DPD method showed that such a coarse-grained model of a surfactant can reproduce self-assembly behavior (e.g., micellar, hexagonal, and lamellar phases) with increasing surfactant concentration.

The fundamental equation of the DPD method is Newton’s equation of motion for a particle subject to three types of forces: conservative, dissipative, and random. Details of the DPD method, including the force formula and its application to generic models, are extensively described elsewhere [38,39,40,45].

### 2.2. Simulation Model and Conditions

**Figure 1:**
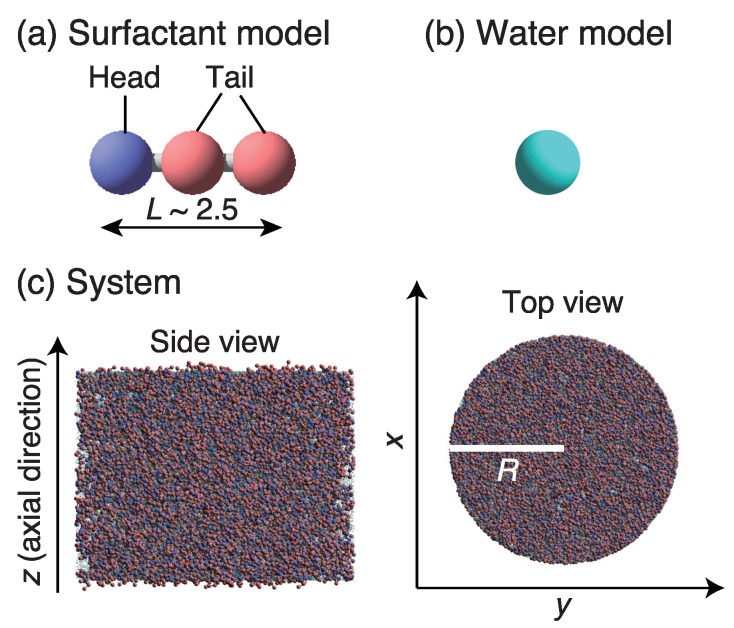
(**a**) Surfactant molecular model composed of one hydrophilic head particle (blue) and two hydrophobic tail particles (red). Length of the surfactant molecule (*L*), calculated from bond length distribution and the radial distribution function, was approximately 2.5 in the DPD dimensionless unit. (**b**) Water molecular model composed of a single particle (aqua). (**c**) Side and overhead (axial) views of tube system. Inner surface of the cylindrical tube was treated as smooth.

We used a surfactant molecular model (Figure 1a) that contained one hydrophilic head (h) particle and two hydrophobic tail (t) particles. The nearest-neighbor particles in the surfactant molecule were connected by harmonic springs. Spring force $F_{ij}^{S}$ between the *i*-th and *j*-th particles (located at $r_{i}$ and $r_{j}$, respectively) is given by

(1) $$F_{ij}^{S}=-k_{s}\left(\right|r_{ij}\left|-r_{s}\right)n_{ij},$$

where $k_{s}$ is the spring constant, $r_{s}$ is the equilibrium bond distance, $r_{ij}=r_{j}-r_{i}$, and $n_{ij}=r_{ij}/\left|r_{ij}\right|$. In this study, values $k_{s}$ = 100$\;k_{B}T/r_{c}^{2}$ and $r_{s}$ = 0.86 $r_{c}$ were adopted, where $r_{c}$ is the cutoff distance. The length of the surfactant molecule calculated from the bond-length distribution and the radial distribution function was approximately 2.5 DPD dimensionless units. The solvent molecular model (Figure 1b) contained a single water (w) particle.

The interaction parameters between any two DPD particles are shown in Table 1. These interactions between any two particles in the solution can be described by the interaction-energy parameters $a_{\mathrm{ww}}=a_{\mathrm{tt}}=a_{\mathrm{wh}}=25\;k_{B}T$, $a_{\mathrm{ht}}=a_{\mathrm{wt}}=70\;k_{B}T$, and $a_{\mathrm{hh}}=40\;k_{B}T$, where w, h, and t represented the water, head group, and tail group, respectively. Hydrophilic and hydrophobic interactions are related to the solubility parameters. For DPD simulations, the interaction (repulsive) parameters between different particles are tuned to reproduce behavior observed in experiments or atomistic simulations. In addition, the interaction parameters for the conservative force between any two particles are related to the Flory–Huggins χ parameters. The choice of these parameters in this study was inspired by the modeling in a previous study of a short surfactant such as cetyltrimethylammonium bromide (CTAB) containing a sodium salicylate (NaSal) solution [46]. This model, with a moderate repulsive force between hydrophilic head groups ($a_{\mathrm{hh}}$ = 40$\;k_{B}T$), can realize both stable threadlike micelle formation and a diffusion coefficient of the surfactant molecules similar to that observed. As the repulsive parameters increase between hydrophilic head groups, the hydration radius may also be estimated to be larger. The same values of interaction parameters were adopted in many studies [43,47,48,49,50,51]. We also examined that our previous bulk simulation of CTAB containing a NaSal solution [47] produced the same results as those in a previous examination that Yamamoto and Hyodo performed [46]. In addition, the surfactant concentration dependence of the self-assembly behavior observed in our previous simulation [43] was consistent with the results of the previously reported experiment [52]. The size (radius and mass) of a single particle has the same value regardless of type [40,53]. The noise amplitude and friction coefficient were set to be 3.0 and 4.5, respectively. The temperature was set at a constant value, i.e., $1.0\;k_{B}T$.

The inner surface of the cylindrical tube was treated as smooth, in agreement with our previous studies [43,48,50,51]. The potential function of the smooth wall was built by summing the DPD force between every solution particle and the wall particles [54]. Integration of this summed force resulted in a force between the DPD particle and the smooth wall (within cutoff distance $r_{c}$). The interaction parameters can be seen as a measure of the magnitude of surface energy. The values of the interaction parameter between the hydrophilic wall surface and water, $a_{\mathrm{wall},w}$, and between the wall and the head group, $a_{\mathrm{wall},h}$, were both set at 25$\;k_{B}T$. The interaction parameter between wall and tail group, $a_{\mathrm{wall},t}$, was set at 70$\;k_{B}T$. The radius (*R*) and length of the tube were 20.0 and 30.0 in dimensionless units, respectively. Density ρ was 5.0; thus, the total number of particles was 188,495. Three surfactant volume fractions (ϕ) were used: 0, 10%, and 30%. The initial configuration for the equilibrium simulations was random (Figure 1c), and a periodic boundary condition was applied in the axial (*z*) direction of the tube.

For generating pipe flow, the virtual density-gradient method [55] was used. When periodic boundary conditions apply in equilibrium simulations, the original cell is typically attached to copies of itself (image cells) at the boundary to resolve the effects of domain surfaces. In this study, the boundary condition was modified by the elongation and contraction of the image cell, producing a density (or pressure) gradient. As a result, pressure-driven flow was generated. Full details of the procedure are given in [55]. For the range of investigated Reynolds numbers Re, the no-slip boundary condition was satisfied, since we considered the wall surfaces to be hydrophilic in this study. Previous experiments showed that the velocity slip depends on surface hydrophilicity [56], and that the velocities near a hydrophilic microchannel wall agree with those predicted by the no-slip boundary condition [57].

## 3. Results and Discussion

To obtain initial configurations for the flow simulations, equilibrium simulations of surfactant aqueous solutions were performed at each volume fraction. At rest, spherical and threadlike micelles were observed at ϕ=0.01 and ϕ=0.03, respectively. These equilibrium morphologies were consistent with those in previous simulation results [50] obtained using a tube model with a 60% smaller radius than the one in this study. Snapshots of these morphologies are shown in Figure S1 of the Supplemental Materials.

**Figure 2:**
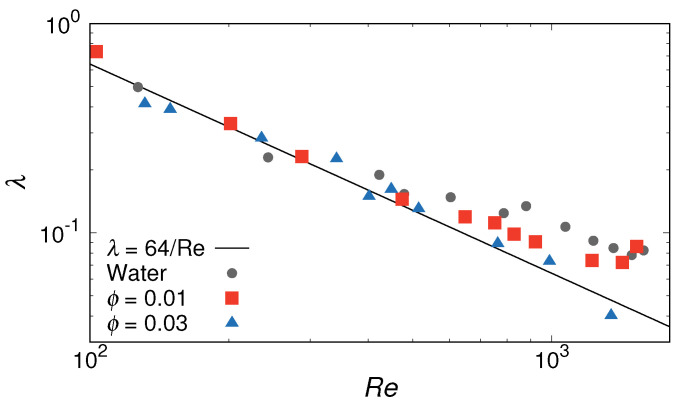
Frictional coefficient of pipe (λ) vs. Reynolds number (Re). Surfactant volume fraction denoted by ϕ. Solid line shows the theoretical estimate for the drag-reduction rate in laminar flow from the Hagen-Poiseuille law. Error bars are smaller than data points.

Figure 2 shows the frictional coefficient λ of the pipe as a function of Re. The volumetric flow rate, *Q*, is estimated by applying the cylindrical shell method to a velocity profile [50,51,58] and a generalized Reynolds number is used [59,60], as we focus on the onset point of the transition to turbulence. Here, the power-law parameter, *n*, is obtained from the relation between the wall shear rate, ${\dot{\gamma }}_{\mathrm{wall}}$, and the wall shear stress, $\tau_{\mathrm{wall}}$, in the steady state (see Figure S2 in the Supplementary Materials). The estimation of flow properties is also described in detail in the Supplementary Materials. For comparison, the theoretical estimate for the drag-reduction rate in laminar flow from the Hagen–Poiseuille law (λ=64/Re) is also shown in the figure. For the pure water case (ϕ=0.00), the λ values were almost in agreement with the theoretical estimates for Re≲250, but they exceeded the theoretical estimate for a laminar flow with Re≳400. The main reason for this discrepancy in the transition from laminar to turbulent flow is the compressibility of the DPD fluid. A previous simulation study [61] reported that the onset of the transition to turbulence shifts to a larger Re as compressibility (Mach number Ma) increases.

**Figure 3:**
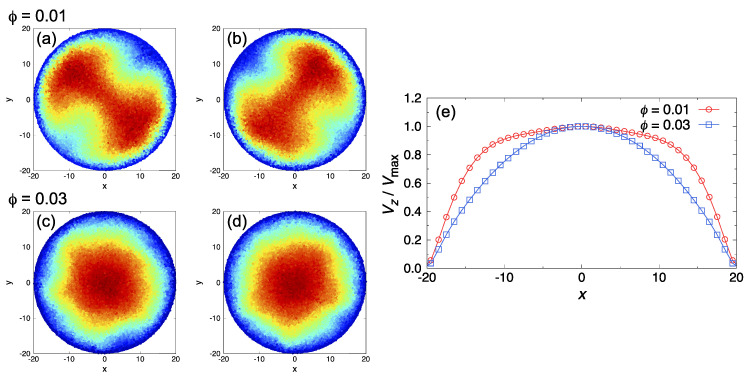
(**a**–**d**) Snapshots of contour maps of streamwise velocity averaged over the pipe length at the highest Re. Blue indicates low-speed streaks, and red indicates high-speed streaks. Two different snapshots (**a**,**b**) for ϕ=0.01 at Re=1526, and (**c**,**d**) for ϕ=0.03 at Re=1344. (**e**) Comparison of velocity profile for the highest Re at each surfactant volume fraction. The vertical axis represents the normalized velocity in the axial (*z*) direction, $V_{z}/V_{\max}$, where $V_{\max}$ is the maximum velocity of the flow.

In this study, we focused on the effect of self-assembled structures of surfactants on the qualitative difference in the onset point of the transition to turbulent flow. When surfactants were added, the transition to turbulence was suppressed for both ϕ=0.01 and ϕ=0.03. For ϕ=0.01, the transition started at a larger Re than that for the pure water case. Further, the frictional coefficients of the pipe at ϕ=0.01 were smaller compared to those in the pure water case for 800≲Re≲1500. When increasing the surfactant volume fraction to ϕ=0.03, there was near agreement between the theoretical estimate of λ in laminar flow and the simulation results over the entire investigated range of Re; a transition to turbulence was not observed. This ϕ dependence of the frictional coefficient was also confirmed in previous molecular-simulation [62] and experimental [63,64,65] studies. A saturation concentration of additives (“Virk’s asymptote” [66]) may appear; however, only two volume fractions of the surfactant were considered in this study. To confirm that the flow was turbulent and not viscoelastic instability, contour maps of the streamwise velocity averaged over the pipe length at the highest Re for both ϕ=0.01 and ϕ=0.03 are shown in Figure 3. Blue indicates low-speed streaks, and red indicates high-speed streaks. At ϕ=0.01, fast streaks were widely distributed in the radial direction, and distribution behavior was changed as time progressed (Figure 3a,b). At ϕ=0.03, contour maps showed typical Poiseuille’s flow, and a steady flow was maintained as shown in Figure 3c,d. We also compared the normalized velocity profile for the highest Re at each surfactant volume fraction, as shown in Figure 3e. It was confirmed the influence of turbulent flow in moving the shear gradients to the edge of the pipes, and found that a flattened velocity profile was obtained, similar to “plug flow” at ϕ=0.01. Thus, these results correspond with the results of λ vs. Re.

**Figure 4:**
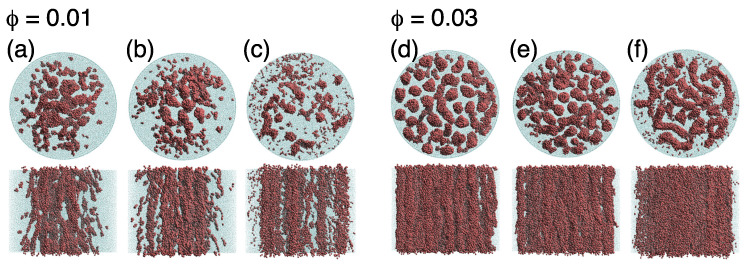
Snapshots of steady-state morphologies of surfactant aqueous solution confined in a hydrophilic tube at volume fractions (**a**–**c**) ϕ=0.01 and (**d**–**f**) ϕ=0.03. (**a**,**d**) Laminar and (**b**,**e**) turbulent regimes correspond to Re below 450 or above 700, respectively; region between these values is (**c**,**f**) the transition state. For clarity, hydrophilic head particles are not shown.

To understand the mechanism of the drag-reduction effect, we next discuss the relation between self-assembly and the transition to turbulent flow. Figure 4 shows representative simulation snapshots of surfactant aqueous solution under pipe flow at ϕ=0.01 (panels (a–c)) and ϕ=0.03 (panels (d–f)). Here, we consider three flow regimes on the basis of the relation between λ and Re at ϕ=0.01. For Re≲450, λ values showed good agreement with the theoretical estimates; this region was defined as the laminar state. For 450≲Re≲700, λ increasingly exceeded the theoretical estimates; this region was defined as the transition state. For Re≳700, the difference in λ between simulation results and theory was approximately constant; this region was defined as the turbulent state. For comparison, the data for ϕ=0.03 were collected at almost the same Re value as for ϕ=0.01.

**Figure 5:**
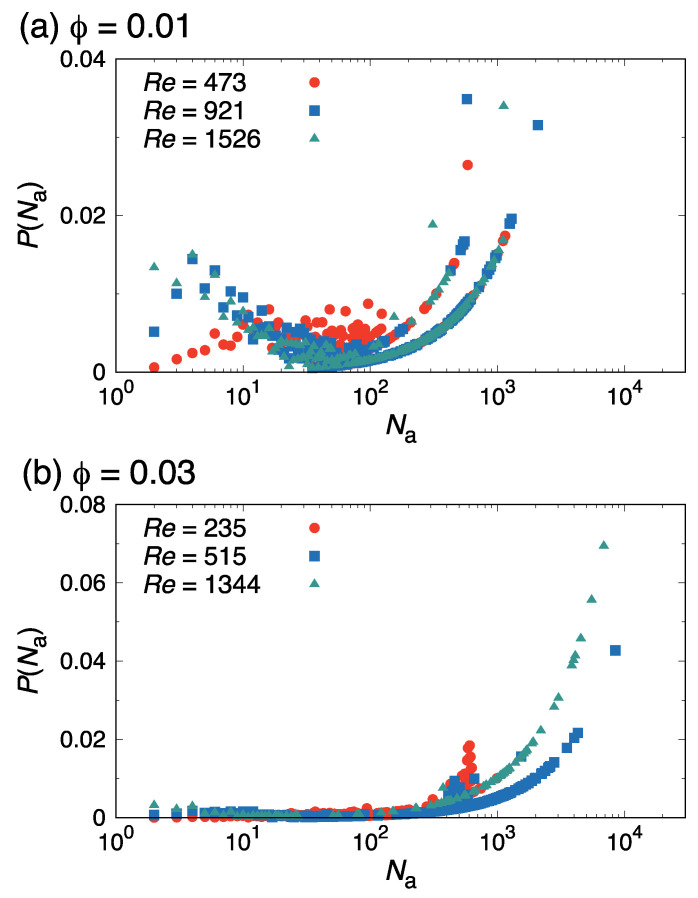
Surfactant cluster size probability distributions $P(N_{a})$ at (**a**) ϕ=0.01 and (**b**) ϕ=0.03 for various Reynolds numbers Re, as indicated.

At ϕ=0.01 in the laminar state, spherical micelles collided with each other and became rodlike, as shown in Figure 4a. This indicates that rodlike micelles maintained laminar flow at higher Re when comparing to the pure water case. Previous studies [29,67,68,69] reported that rodlike micelles are needed for drag reduction; our results support this. For the transition state (450≲Re≲700), cluster-size probability distribution $P(N_{a})$, shown in Figure 5a, showed that flow-enhanced collisions caused the growth of micelles (cluster size $N_{a}≳10^{3}$) to be formed more than that in the laminar state, but a peak appeared in the distribution in the $1≲N_{a}≲10$ range. As the Reynolds number further increased, the probability of $N_{a}=1$ (i.e., of monomers) increased, and the peak of $P(N_{a})$ shifted to lower $N_{a}$ values. Thus, as flow became completely turbulent, micelles became smaller and broke up into monomers. These results suggest that the number and formation of micelles are closely related to the suppression of the turbulent transition. Drag-reduction phenomena depend on the diameter of the tube. Many previous studies [70,71,72,73,74] reported that the tube diameter has an inverse effect on drag-reduction rate. When the tube diameter was increased, larger eddies that cause energy loss were observed. Therefore, in this sense, since the length scale ratio of the micelles to the turbulent eddy size was also a significant factor, it was assumed that the necessary conditions for obtaining the drag-reduction effect that we presented had some impact, even at the same surfactant volume fractions.

For a more concentrated system (ϕ=0.03) with a relatively low Re≲450 (corresponding to the laminar state when ϕ=0.01), threadlike micelles were oriented along the flow (*z*) direction, as shown in Figure 4d. When the Reynolds number increased to Re≈700 (corresponding to the transition state when ϕ=0.01), the shape of the micelles remained unchanged, as shown in Figure 4e. In contrast to the dilute case (ϕ=0.01), only the flow-induced growth of the rodlike micelles appeared; the increase in monomers was not observed (see Figure 5b). For Re≳700 (corresponding to the turbulent state when ϕ=0.01), the rodlike micelles grew further and eventually became sheet-shaped (see top view in Figure 4f). For more quantitative information, we calculated the radius of gyration of a micelle G with corresponding eigenvalues $G_{1}\geq G_{2}\geq G_{3}$, and then computed the relative shape anisotropy parameter ($\kappa^{2}$), defined as $\kappa^{2}$ is given by

(2) $$\kappa^{2}=1-3\frac{G_{1}G_{2}+G_{2}G_{3}+G_{3}G_{1}}{{\left(G_{1}+G_{2}+G_{3}\right)}^{2}}.$$

**Figure 6:**
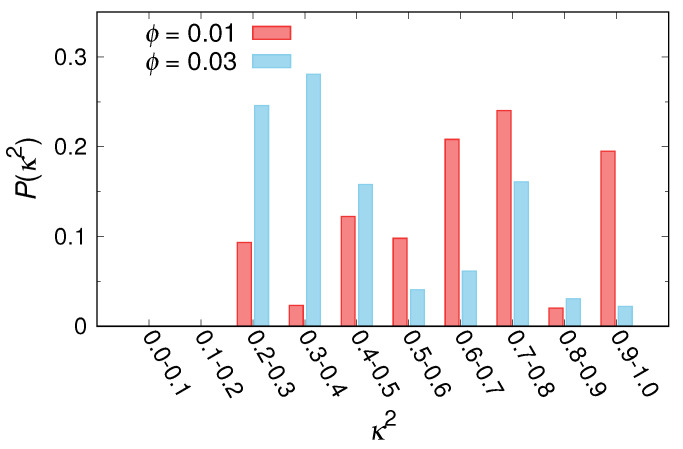
Comparison of relative shape anisotropy parameter ($\kappa^{2}$) distributions for micelles under turbulent flow with different volume fractions, ϕ.

This parameter was bounded between the values of 0 and 1, which corresponded to perfect spherical and linear shapes, respectively. Figure 6 shows the comparison of the relative shape anisotropy parameter ($\kappa^{2}$) distributions for micelles under turbulent flow with different volume volume fractions, ϕ. For ϕ=0.01, the distribution at $\kappa^{2}=0.7–0.8$ was the largest, and the distribution at $\kappa^{2}=0.9–1.0$ that indicated the existence of rodlike micelles was also relatively large. In contrast, for ϕ=0.03, $\kappa^{2}$ distribution shifted towards lower values, and a clear decrease in the distribution occurred at $\kappa^{2}=0.9–1.0$. Thus, although the size and shape of micelles changed in the range of Re≳700, the turbulent transition was still effectively suppressed (Figure 2).

**Figure 7:**
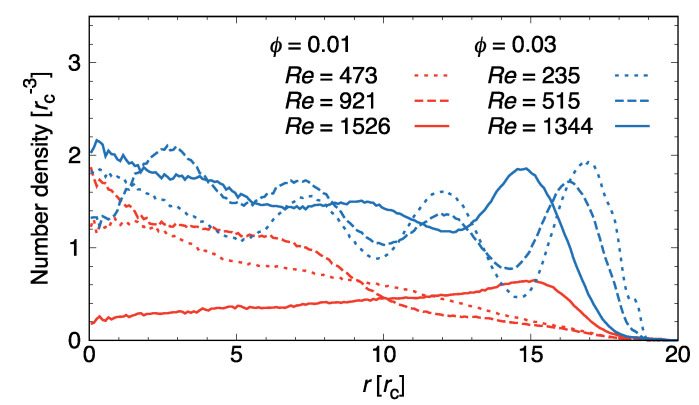
Density profiles of surfactant molecules in radial direction. Re: Reynolds number; ϕ: surfactant volume fractions.

To investigate in detail why a ϕ-dependent delay in the turbulent transition occurred, we computed the density profiles of surfactant molecules in the radial direction in the steady state (Figure 7). For ϕ=0.01, we found a distinct difference in density profiles between the turbulent state and the others. For the laminar and transition states, surfactant molecules were distributed within the central region of the tube (r<10), as an adequate number of rodlike micelles still existed. By contrast, the peak of the density profile shifted to r≈15 for the turbulent state. Thus, for dilute systems, the drag-reduction effect disappeared due to large micelles breaking up into smaller ones and eventually into monomers.

For dense systems, there was also a difference in density profiles, particularly for the turbulent state. For Re≲700, several peaks could be seen in the radial direction, reflecting the distribution of orientationally ordered rodlike micelles along the flow direction over the entire radial range. When Re was increased over 700 (corresponding to the turbulent state with ϕ=0.01), distinct peaks in the range of r<15 disappeared as a result of rodlike micelles changing into sheet-shaped ones.

## 4. Conclusions

We presented a possible molecular-level mechanism of turbulence suppression in surfactant aqueous solutions based on a large-scale dissipative particle dynamics simulation. Our simulations revealed that the phenomenon of the drag-reduction effect was caused by turbulence suppression, and the number and formation of micelles were closely related to the suppression of the turbulent transition. We established the necessary conditions for obtaining the drag-reduction effect: maintaining (i) a certain minimal size of micellar structures ($N_{a}≳10^{3}$), even at high Re; and (ii) a homogeneous distribution of surfactant molecules in the radial direction of the tube. To the best of our knowledge, our work is the first to show molecular-simulation evidence for the relation between micellar structure and drag reduction in pipe flow. Our findings provide new insights into the mechanism of drag reduction on the molecular level, and may prove valuable for identifying the required synthesis to obtain the drag-reduction effect in a targeted range of Reynolds numbers.

## Figures and Tables

**Table 1:** Interaction parameters $a_{ij}$ (in $k_{B}T/r_{c}$ units) between all pairs.

	h	t	w	Wall
**h**	40	70	25	25
**t**	70	25	70	70
**w**	25	70	25	25
**Wall**	25	70	25	–

## Data Availability

Not applicable.

## Supplementary Materials

The following are available online at https://www.mdpi.com/article/10.3390/ijms22147573/s1.

## Abbreviations

The following abbreviations are used in this manuscript:

DPD	Dissipative particle dynamics
CTAB	Cetyltrimethylammonium bromide
NaSal	Sodium salicylate
