# Supplementary material for: Molecular Insight into the Possible Mechanism of Drag Reduction of Surfactant Aqueous Solution in Pipe Flow
Source: Int J Mol Sci. 2021 Jul 15;22(14):7573. doi: 10.3390/ijms22147573 (PMC8307477; doi:10.3390/ijms22147573)
Supplement: Supplementary file 1 [file ijms-22-07573-s001.zip › ijms-1271466-supplementary.pdf]

Molecular Insight into the Possible Mechanism of Drag Reduction of Surfactant Aqueous Solution in Pipe Flow

Yusei Kobayashi,* Hirotaka Gomyo, and Noriyoshi Arai

Department of Mechanical Engineering, Keio University, 3-14-1 Hiyoshi, Kohoku-ku, Yokohama 223-8522, Japan

E-mail: kobayashi@mech.keio.ac.jp

Equilibrium morphologies

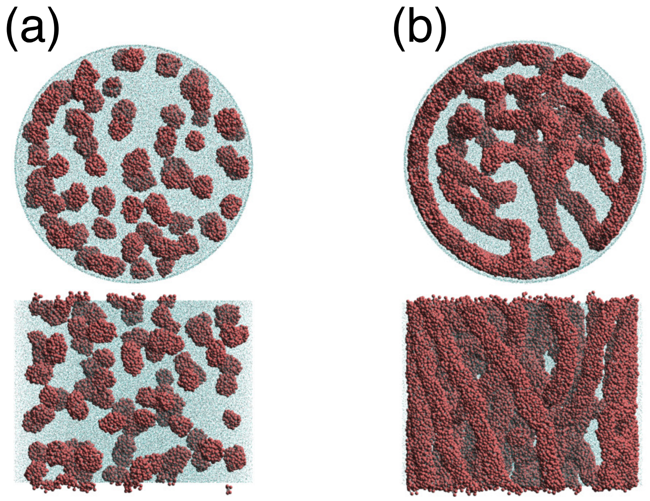

Figure S1: Snapshots of equilibrium morphologies of the surfactant aqueous solution confined in a hydrophilic tube for fractions (a) $\phi = 0.01$ and (b) $\phi = 0.03$. For clarity, hydrophilic head particles are not shown.

Estimation of flow properties

The frictional coefficient λ of a pipe is given by the Darcy-Weisbach formula

$$\lambda = \frac{2\Delta p D}{L\rho v_\mathrm{a}^2} \quad , \tag{1}$$

where Δp is the pressure loss, D is the tube diameter, L is the tube length, ρ is the density, and v_a is the mean velocity of the fluid. Here, v_a is defined as the volumetric flow rate, Q, divided by the cross-sectional area, $A = \pi(D/2)^2$. Q can be estimated by applying the cylindrical shell method to a velocity profile. We use a seventh-degree polynomial function to fit the curve of the velocity profile. The volumetric flow rate obtained by rotating about the axial (z) direction of the tube is then

$$Q = \int_0^R 2\pi r |U_z(r)| dr \quad , \tag{2}$$

where $U_z(r)$ is the velocity profile and r is the radial distance from the center of the tube.

In this study, a generalized Reynolds number Re is used, as we focus on the onset point of the transition to turbulence. The generalized Reynolds number, which expresses the ratio of inertial forces to viscous forces, is defined as

$$Re = \frac{\rho {v_\mathrm{a}}^{2-n} D^n}{K} \quad , \tag{3}$$

where n is the power-law parameter and K is the flow-consistency index. In Figure S2, we plot the relation between the wall shear rate, $\dot{\gamma}_\mathrm{wall} = 4Q/(\pi R^3)$, and the wall shear stress, $\tau_\mathrm{wall} = R\Delta p/(2L)$; we obtain n from the slope. K is defined as

$$K = \left\{ \frac{n\pi R^2}{(3n+1)Q} \right\}^n \left(\frac{R\Delta p}{2L} \right) \quad . \tag{4}$$

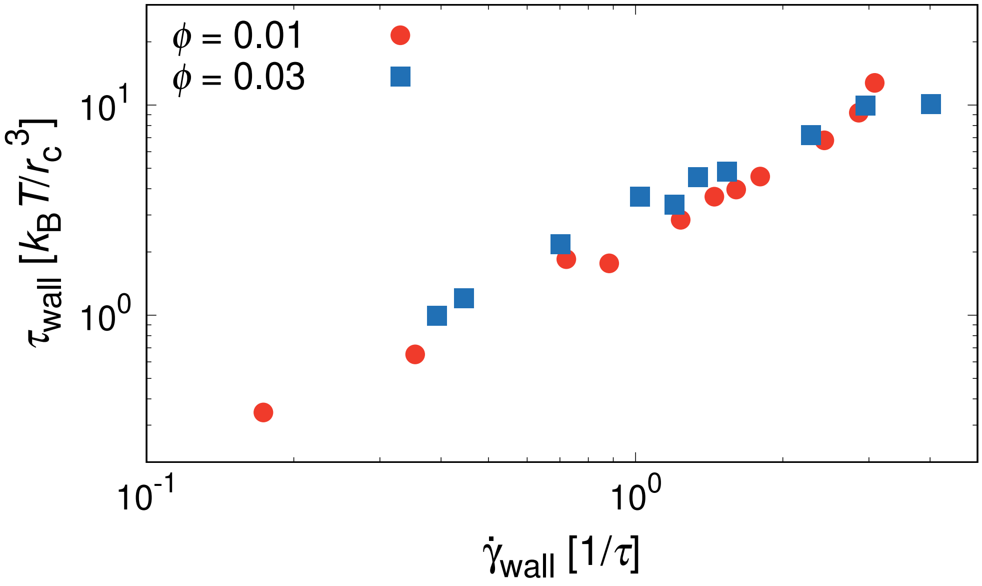

Figure S2: Wall shear rate $\dot{\gamma}_{\mathrm{wall}}$ *vs.* wall shear stress τ_{wall} in the steady state, for different surfactant fractions ϕ.
